# Supplementary material for: Dual role of Ninjurin-1 in myeloid cell adhesion and inflammation in relapse-remitting EAE
Source: Front Immunol. 2026 Apr 15;17:1803382. doi: 10.3389/fimmu.2026.1803382 (PMC13124630; doi:10.3389/fimmu.2026.1803382)
Supplement: Supplementary file 1 [file DataSheet1.pdf]

SUPPLEMENTAL INFORMATION

Dual Role of Ninjurin-1 in Myeloid Cell Adhesion and Inflammation in Relapse-Remitting EAE

Coleen Thompson[1], Alex Annett[1], Anastasia Alkhimovitch[2], Kashish Singh Parihar[2], Igal Ifergan[1,2,3]

FIGURE LEGENDS

Figure S1. Gating strategy for CNS immune cells and Ninjurin-1 detection using isotype control. (**A**) CNS single-cell suspensions were first gated by forward and side scatter (FSC/SSC; P1), followed by exclusion of doublets and dead cells. Infiltrating myeloid cells were identified as $CD45^{hi}$ $CD11b^{+}$ and infiltrating lymphoid cells as $CD45^{hi}$ $CD11b^{-}$. Within the lymphoid compartment, $CD3^{+}$ T cells were further defined. (**B**) Ninjurin-1 expression was assessed on infiltrating myeloid cells and $CD3^{+}$ T cells. Representative plots show isotype control staining (black) and Ninjurin-1 staining (red).

Figure S2. Increased activation and cytokine expression by Ninjurin-1^{+} myeloid cells in the spleen. Comparison of Ninjurin-1^{-} (closed circles) and Ninjurin-1^{+} (open squares) $CD45^{hi}$ $B220^{-}$ $CD11b^{+}$ myeloid cells isolated from the spleen at onset of RR-EAE. Flow cytometry analysis revealed that (**A**) co-stimulatory molecules CD80 and CD86, as well as MHC II, were significantly increased on Ninjurin-1^{+} cells. (**B**) Expression of IL-1β, IL-6, IL-12p40, IL-23p19, TGF-β, and TNF-α was also elevated in Ninjurin-1^{+} myeloid cells ($n = 6$ mice; *$p < 0.05$, **$p < 0.01$, ***$p < 0.001$ by paired t-test).

Figure S3. Cell sorting of $CD45^{+}CD11b^{+}B220^{-}CD3^{-}Ly6G^{-}$Ninjurin-1^{+} and Ninjurin-1^{-} myeloid cells. (**A**) Splenocytes were first enriched for $CD11b^{+}$ cells using Miltenyi magnetic microbeads, followed by sorting on a Sony MA900 cell sorter. At the FACS, cells were sequentially gated by forward and side scatter (FSC/SSC), single-cell discrimination, and viability. Myeloid cells were identified as $CD45^{+}CD11b^{+}CD3^{-}B220^{-}$,

with Ly6G$^+$ neutrophils excluded. (**B**) Ninjurin-1+ and Ninjurin-1$^-$ populations were defined using an isotype control to establish gating thresholds.

Figure S4. Ninjurin-1 blockade reduces the number of cytokine-producing CNS CD4$^+$ T cells without major changes in cytokine frequencies. CNS mononuclear cells were isolated at day 32 post-immunization from RR-EAE mice treated with scramble control or anti-Ninj$_{26\text{-}37}$. Cells were stimulated for 4 hours with PMA and ionomycin in the presence of brefeldin A and analyzed by intracellular cytokine staining. (**A**) Frequency of IL-17$^+$, IFN-γ$^+$, GM-CSF$^+$, TNF-α$^+$, and TGF-β$^+$ cells among CNS CD4$^+$ T cells. (**B**) Absolute numbers of cytokine-producing CD4$^+$ T cells in the CNS. Each dot represents one mouse (n = 3 per group). Bars indicate mean ± SEM. Data are representative of one experiment. Statistical analysis was performed using two-way ANOVA (*p < 0.05, **p < 0.01, ***p < 0.001).

Figure S1

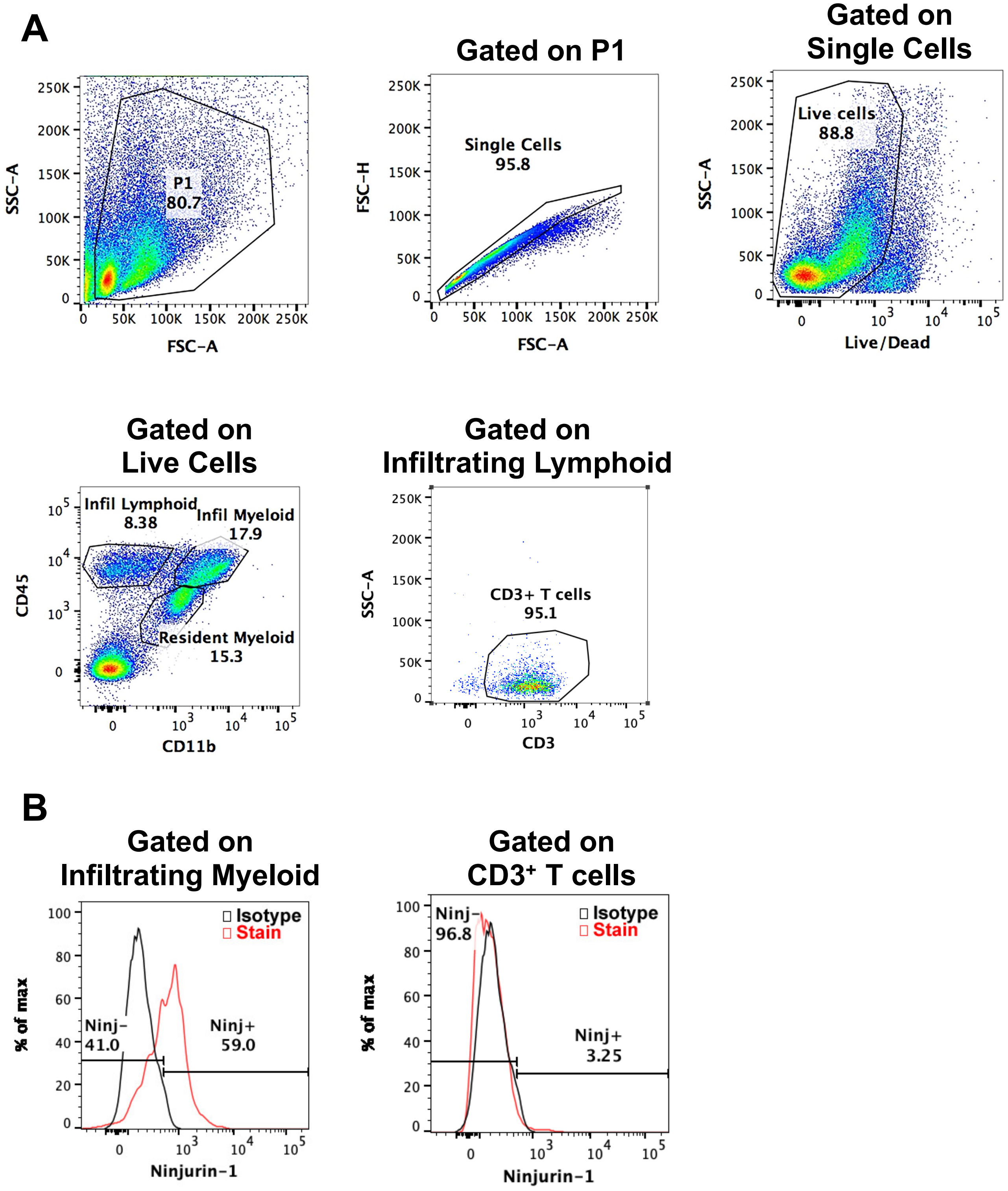
A
Gated on P1
Gated on Single Cells
P1
80.7
Single Cells
95.8
Live cells
88.8
SSC-A
FSC-A
FSC-H
Live/Dead
Gated on Live Cells
Gated on Infiltrating Lymphoid
Infil Lymphoid
8.38
Infil Myeloid
17.9
Resident Myeloid
15.3
CD45
CD11b
CD3+ T cells
95.1
CD3
B
Gated on Infiltrating Myeloid
Gated on CD3+ T cells
Isotype
Stain
Ninj-
41.0
Ninj+
59.0
% of max
Ninjurin-1
Ninj-
96.8
Ninj+
3.25

Figure S2

A

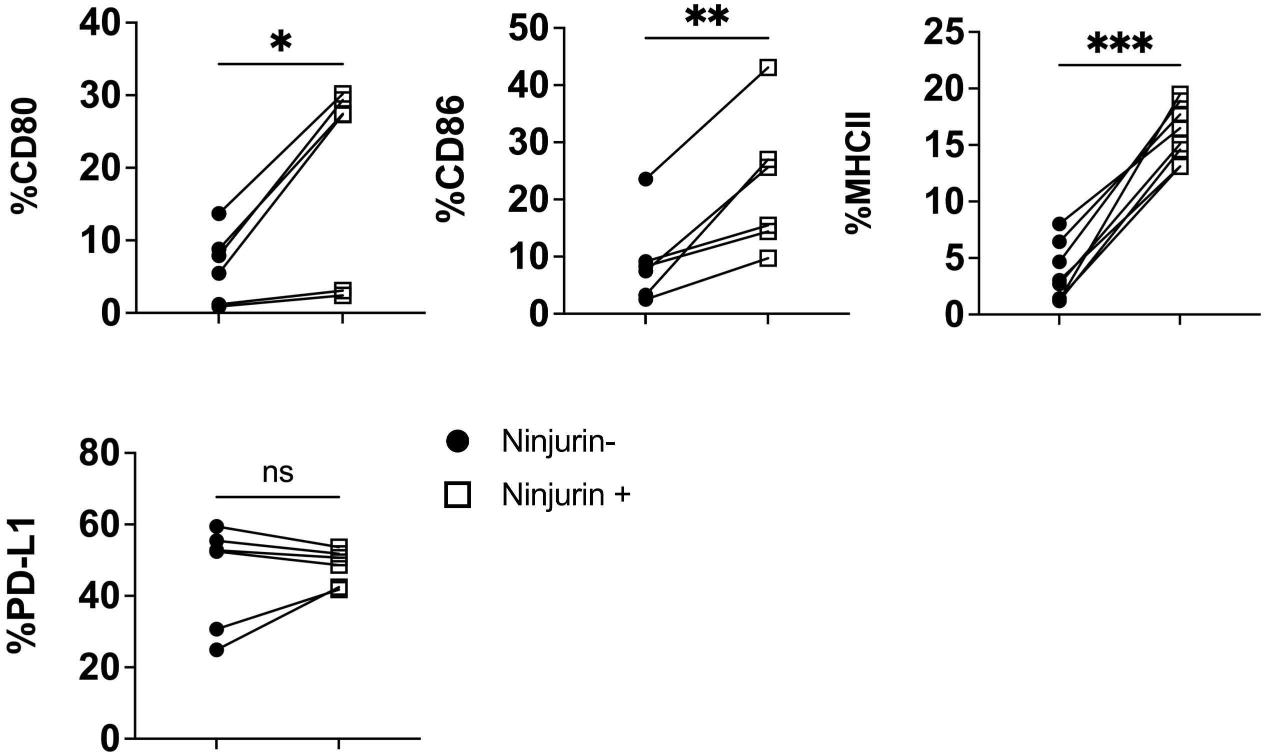

%CD80
*
%CD86
**
%MHCII
***
%PD-L1
ns
Ninjurin-
Ninjurin +

B

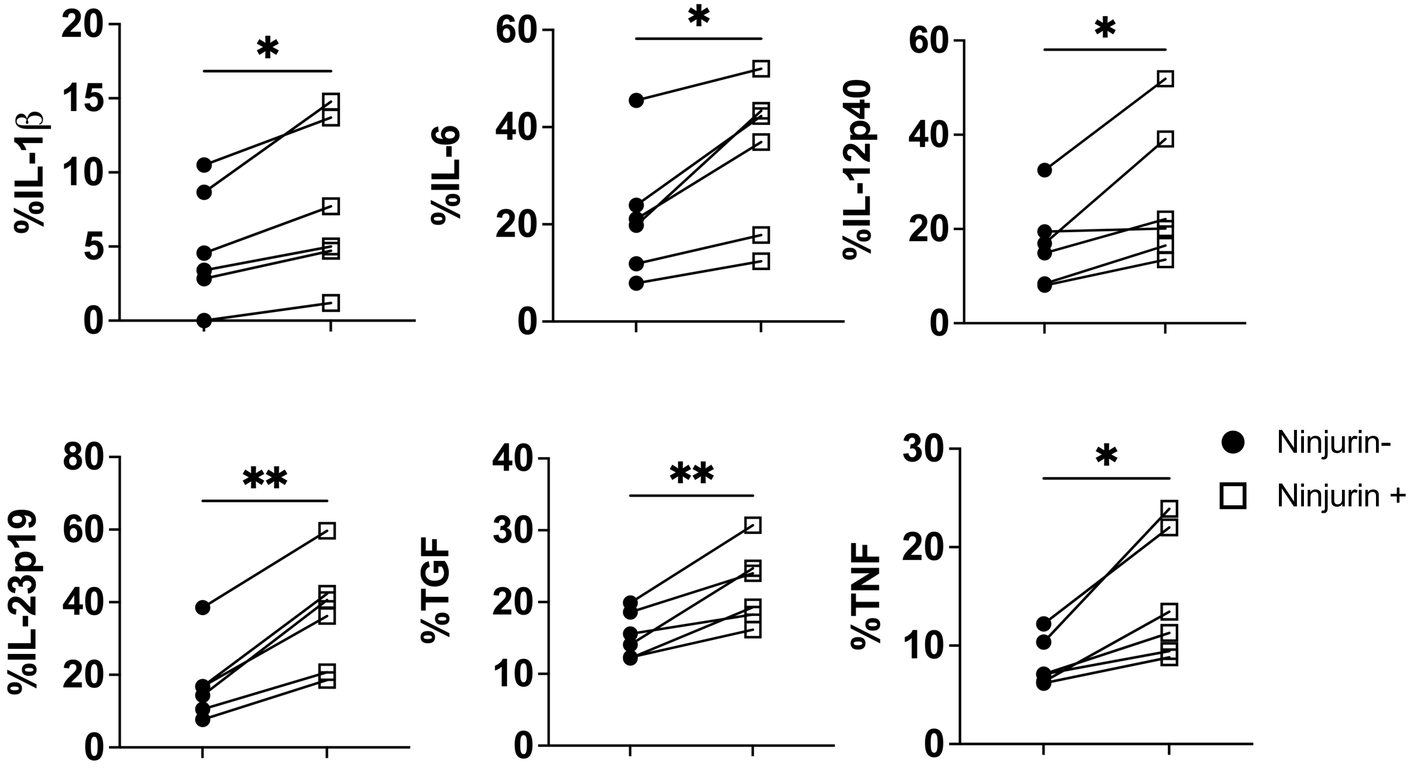

%IL-1β
*
%IL-6
*
%IL-12p40
*
%IL-23p19
**
%TGF
**
%TNF
*
Ninjurin-
Ninjurin +

Figure S3

A

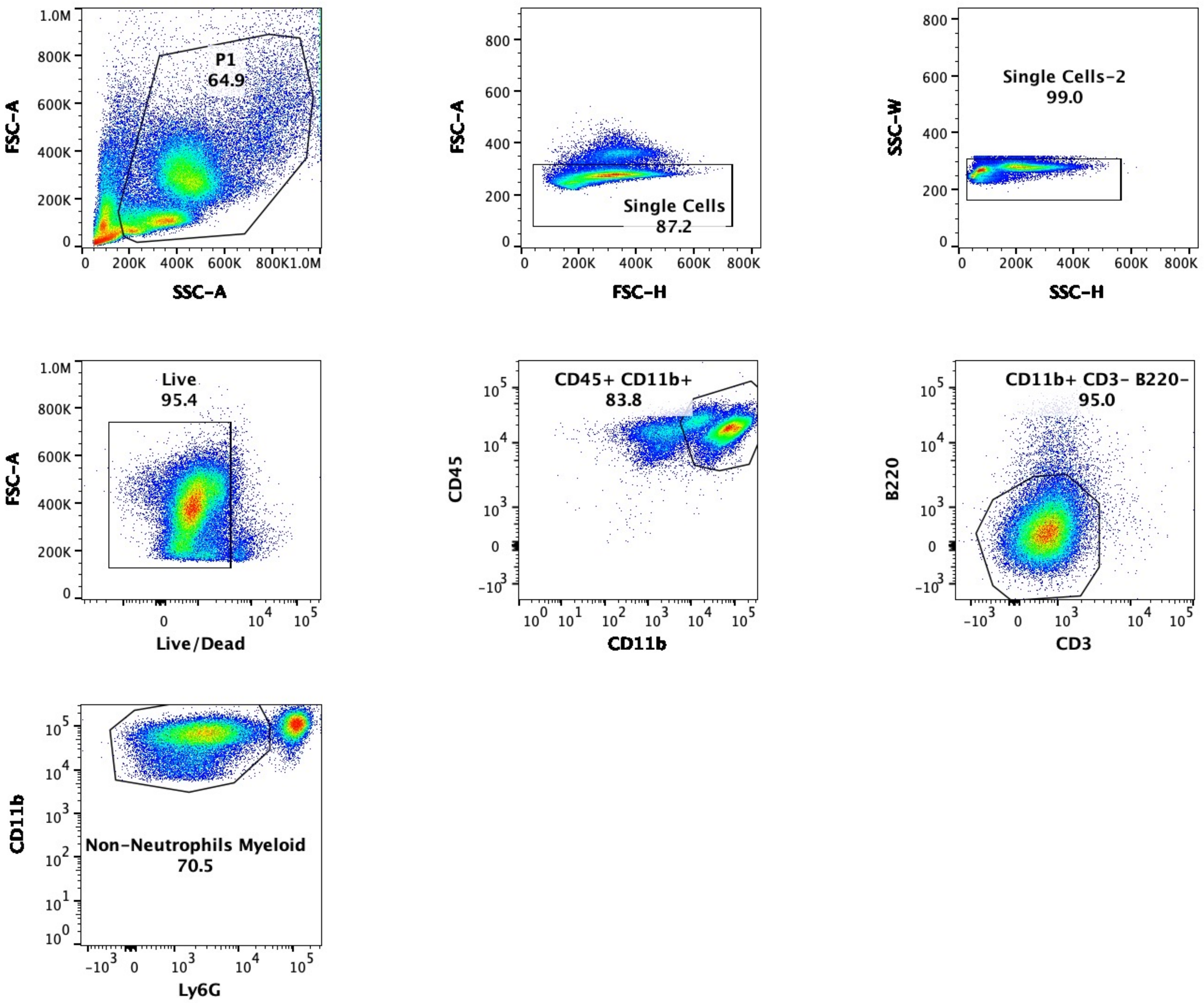

P1
64.9
FSC-A
SSC-A
Single Cells
87.2
FSC-H
Single Cells-2
99.0
SSC-W
SSC-H
Live
95.4
Live/Dead
CD45+ CD11b+
83.8
CD45
CD11b
CD11b+ CD3- B220-
95.0
B220
CD3
Non-Neutrophils Myeloid
70.5
Ly6G

B

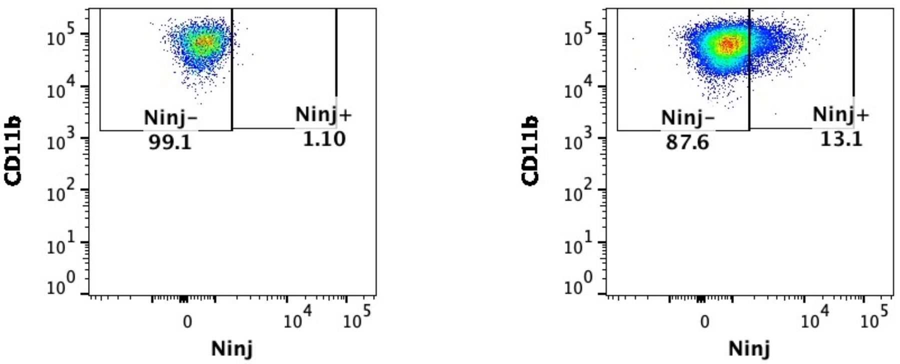

Ninj-
99.1
Ninj+
1.10
CD11b
Ninj
Ninj-
87.6
Ninj+
13.1
CD11b
Ninj

Figure S4

A

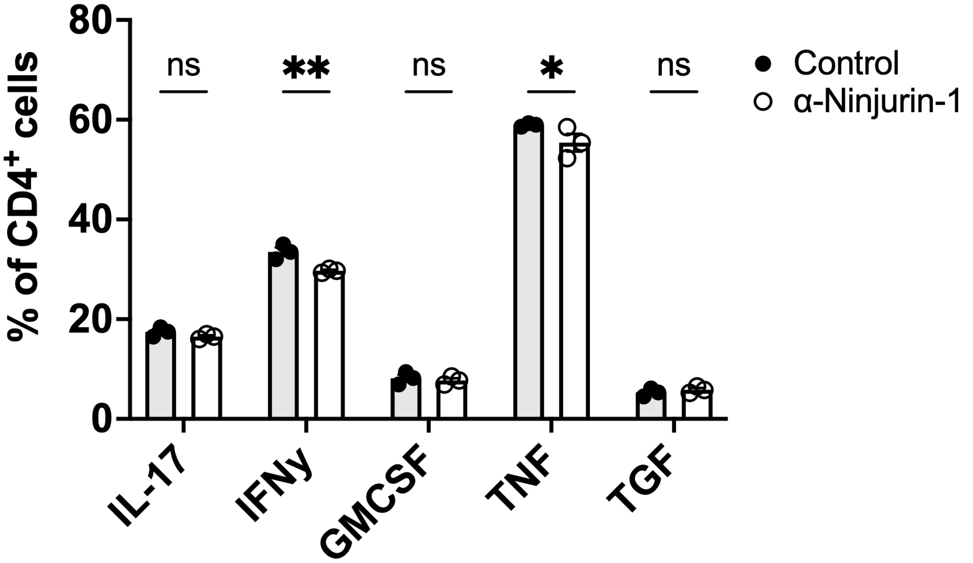

80
60
40
20
0
% of CD4+ cells
ns
**
ns
*
ns
Control
α-Ninjurin-1
IL-17
IFNy
GMCSF
TNF
TGF

B

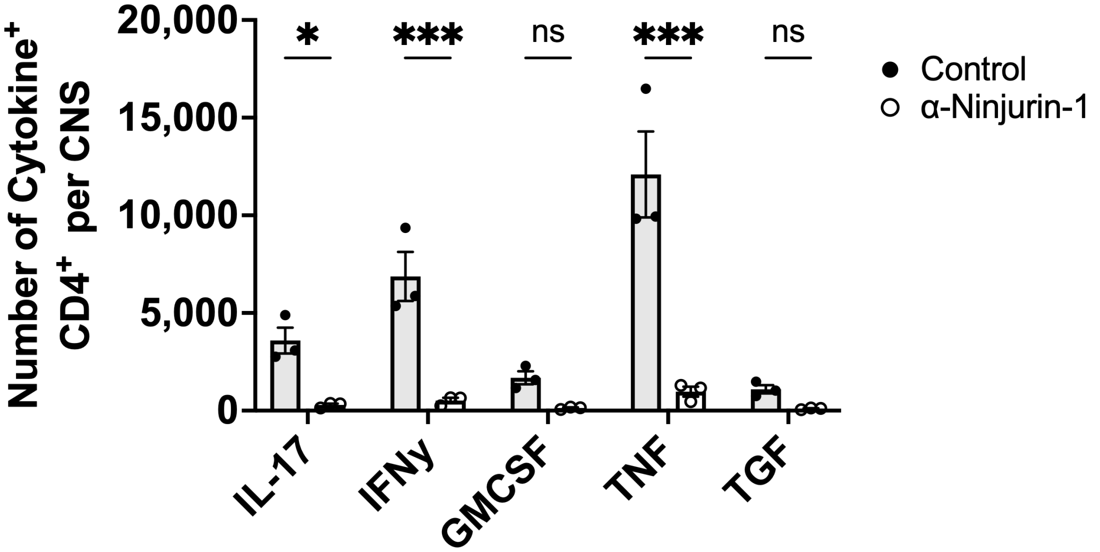

20,000
15,000
10,000
5,000
0
Number of Cytokine+ CD4+ per CNS
*
***
ns
***
ns
Control
α-Ninjurin-1
IL-17
IFNy
GMCSF
TNF
TGF
